# Supplementary material for: The dark side of streaking: Examining the backfire potential of run streaking in recreational runners who broke a long-term streak
Source: PLoS One. 2026 May 22;21(5):e0317254. doi: 10.1371/journal.pone.0317254 (PMC13196939; doi:10.1371/journal.pone.0317254)
Supplement: S1 File — (DOCX) [file pone.0317254.s001.docx]

**Appendix A - Topic Guide**

**Part 1: Running and streaking in general**

Let’s start with some questions about your running in general and your streak running.

1. **For how long have you been running overall, not just run streaking?**
2. **Besides running, do you do any other types of physical activity at the moment?**
   1. Have you always been running as an activity, or have you done other types of activity in the past?
3. **For run streaking specifically, how long have you been doing it?**
   1. Are you currently still streaking?
   2. [If still streaking] What was your longest ever streak? Is that your current streak?
4. **How did you get into run streaking?**
   1. Was it planned or accidental?
5. **Do you think run streaking is a useful strategy for everyone who want to be physically active?**
   1. If so, why and for whom?
6. **What are some of the positives of run streaking?**
   1. Routine
   2. Accomplishment
   3. Community
   4. Fitness
7. **What might be some of the negatives of run streaking?**
   1. Injury
   2. Impact on social life
   3. Stress
   4. Time and energy
   5. Costs of equipment
8. **How did you cope with some of the negative consequences of streaking?**
9. **Were there times where you felt like you were obsessed with your streak?**
10. **Did maintaining your streak ever feel like a burden?**

**Part 2: Streak breaking or retiring the streak**

In this second part I would like to ask you a few questions about the time when you stopped your run streak. Here I am interested in the last streak that stopped which was longer than 100 days?

1. **Did you stop your streak intentionally or was it not intentional?**
   1. *If intentional*: What led you to this decision? Was this a sudden decision, or was it a process?
   2. *If non-intentional*: What was the reason why they had to stop?
      1. Injury or surgery, Life event, Job or family commitment
2. **Can you remember the actual day that your streak stopped?**
   1. [If yes] Could you tell me about what happened that day?
3. **How did you feel after your streak stopped?**
   1. The next day, weeks, month
4. **How did your life change after stopping your streak?**
   1. What was your relationship with physical activity like?
   2. What did you do with your free time?
   3. How did it affect your social life?
5. **Did you continue to run after your streak ended?**
   1. [If yes] When did you start again? How frequently did you run?
   2. [If no] Did you do any other type of physical activity instead?
6. **Did you start another streak? Why/why not?**
   1. [If yes] When did you decide that you will start another streak? How much time was there between the end of streak and the beginning of your next streak?
7. **What lessons have you learnt from run streaking?**
   1. About yourself, physical activity, accomplishing goals
8. **What else you would like to tell us about streaking that we haven’t asked about?**

Thank you for answering all my questions. We really appreciate you participating in this research study. Finally, I would like to ask you some brief questions about yourself.

1. **Demographic Questions.**
   1. How old are you?
   2. How would you describe your gender?
   3. What is your relationship status?
   4. Height/ Weight?
   5. How would you describe your ethnicity?
   6. What is your employment status?
